# Novel Calibration Approach for Monitoring Aerosol Hydrogen Isotopes Using Laser-Induced Breakdown Spectroscopy for Molten Salt Reactor Off-Gas Streams

**DOI:** 10.3390/s23249797

**Published:** 2023-12-13

**Authors:** Hunter B. Andrews, Joanna McFarlane

**Affiliations:** 1 Radioisotope Science and Technology Division, Oak Ridge National Laboratory, 1 Bethel Valley Road, Oak Ridge, TN 37831, USA; 2 Nuclear Energy and Fuel Cycle Division, Oak Ridge National Laboratory, 1 Bethel Valley Road, Oak Ridge, TN 37831, USA

**Keywords:** laser-induced breakdown spectroscopy (LIBS), isotopic measurements, protium, deuterium, tritium, molten salt reactors (MSRs), online monitoring

## Abstract

Online monitoring is a key challenge for the continued development of molten salt reactor (MSR) technology. Laser-induced breakdown spectroscopy (LIBS) has previously been demonstrated to be a viable tool for monitoring aerosolized species and noble gases in real time, but the ability to discern varying isotopes in these streams has not yet been investigated for MSR applications. Tritium will form in MSRs from ternary fission and from (n,α)-reactions occurring in lithium-containing salts. This study compares three spectrometers of varying resolutions and types for measuring hydrogen isotope shifts in LIBS spectra of wetted filter paper. For each spectrometer, multivariate models were built (i.e., principal component regression, partial least squares regression, and multivariate curve resolution) to quantify the isotope ratio. The top models were then modified and corrected to apply the models to aerosol samples with varying isotope ratios. This novel calibration strategy offers an 82% reduction in volume of the calibration samples needed and is a more viable pathway for calibrating deployable LIBS systems. Lastly, this calibration model was compared with an all-aerosol trained model for monitoring hydrogen isotopes during a real-time test where the protium/deuterium ratio, along with representative salt species (i.e., lithium, sodium, and potassium) were adjusted dynamically. Results of this test validated the predictive capabilities of the transferred model and highlighted the capabilities of LIBS for real-time monitoring of MSR effluent streams.

## 1. Introduction

Molten salt reactors (MSRs) are complex systems with system-wide chemistry challenges differing from their solid-fueled, light-water reactor cousins. MSRs consist of a primary reactor loop containing either fluoride or chloride salt mixtures with dissolved fissile fuel. This unclad fuel will undergo nuclear fission in the reactor, producing heat and fission products, some of which will readily evolve from the salt. Other species may be produced through radioactive decay or subsequent nuclear reactions (e.g., neutron absorbance), leading to further species, which could either be volatile or migrate into the reactor headspace through aerosolization. An off-gas treatment system is vital to the operation of MSRs, serving to remove these products, properly confine them, and maintain an inert environment for the fuel salt [1].

In recent years, several studies have been performed in an organized effort to establish off-gas treatment components and online monitoring capabilities. Briefly, proof-of-principal, real-time monitoring of aerosols and noble gases has been performed using laser-induced breakdown spectroscopy (LIBS) [2,3,4,5]. LIBS has been used to evaluate the performance of metal–organic frameworks for selectively capturing xenon as a demonstration of in situ monitoring of off-gas components [6]. Raman spectroscopy systems have been developed to monitor iodine species [7]. Additionally, Raman and ultraviolet–visible absorbance spectroscopies have been used in tandem to simultaneously monitor salt and gas-phase compositional changes [8].

One capability of LIBS that has yet to be demonstrated for an MSR-relevant system is the ability to discern isotopic ratios. Although LIBS is used primarily as an elemental technique, for specific elemental and molecular emissions, a wavelength shift can be measured and modeled to evaluate isotope contributions. In the last decade, a significant effort in the LIBS community has revolved around isotopic measurements via LIBS and its extension, laser ablation molecular isotopic spectroscopy (LAMIS) [9,10,11,12,13,14,15]. LIBS isotope measurements are performed using shifts in the emission peaks; LAMIS uses peak shifts of molecular emissions, which occur later in the plasma lifetime but present larger shifts [10].

Tritium is of significant interest in MSRs because it is especially permeable. It will be continuously produced during operation through ternary fission and (n,α)-reactions in salts containing lithium [1]. Hydrogen, being the lightest element in the periodic table, experiences the largest relative mass difference between its isotopes: protium, deuterium, and tritium. This difference, in turn, means the H_α_ Balmer line at 656 nm, the most intense hydrogen emission, experiences a significant isotopic peak shift [12]. Hydrogen isotopes have been monitored with high accuracy in the gas phase using LAMIS, but in the case of MSR process streams, the O–H bands would likely have interferences from many of the salt and fission product species [9]. The ability to simultaneously monitor tritium and fission product migration in real time via LIBS could offer a feasible way for reactor operators to better understand the state of the fuel salt in the reactor, informing decisions such as when to process the salt.

The objective of this work is to demonstrate the use of LIBS for monitoring hydrogen isotopes in an MSR-relevant system (e.g., an aerosolized matrix). Three points of scientific advancement are covered in this work: (1) investigate several different spectrometer types for their ability to measure the peak shift of the 656 nm hydrogen line, (2) compare various multivariate models (i.e., principal component regression [PCR], partial least squares regression [PLSR], and multivariate curve resolution [MCR]) predictive capabilities for each spectrometer system, and (3) demonstrate a novel approach to calibrating a LIBS isotope shift model by transferring a calibration performed on one matrix to another, resulting in significant resource savings.

## 2. Experimental

### 2.1. Sample Preparation

Various ratios of D_2_O (99.99%, Sigma-Aldrich, St. Louis, MO, USA) and milliQ H_2_O (18 MΩ·cm) were loaded into sample vials using a 20 µL pipette. The vials were shaken vigorously to ensure that the liquids mixed. Samples ranging from 0% to 99.9% D_2_O in increments of 10% were prepared, resulting in 11 samples. Here, deuterium was used as a surrogate for tritium to minimize radiological hazards. For initial tests, 10 µL of each sample was pipetted onto a 5 mm punch-out of a Whatman filter paper (1541-047, Cytiva, Marlborough, MA, USA) affixed to a glass microscope slide using double-sided carbon tape. The 10 µL of sample was selected because it fully saturated the filter without excess liquid. The samples were immediately placed into the LIBS system for testing before any liquid could evaporate.

### 2.2. LIBS Filter Sample Tests

**Figure 1:**
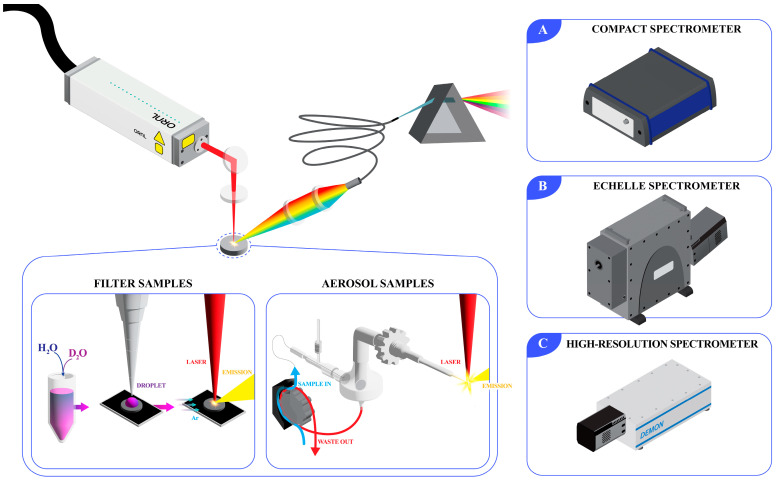
Diagram of experimental LIBS setup showing measurements using a (**A**) compact, (**B**) echelle, and (**C**) high-resolution spectrometer on filter samples or an aerosol stream.

The LIBS system used was a LIBS-8 module (Applied Photonics, Skipton, UK) with a 1064 nm Nd:YAG laser at 10 Hz. Filter sample measurements were performed with 25 mJ for the compact and high-resolution spectrometers, whereas the echelle spectrometer measurements were made with 100 mJ to provide similar signal-to-noise levels. The system allowed for the sample and optical pathway to be purged with high-purity argon (99.999%, AirGas, Radnor, PA, USA) to prevent detecting atmospheric hydrogen in the collected spectra. The laser was focused onto the sample surface to a spot size of 100 µm. The plasma light was collected at a slight angle from the incident laser pulse. For the filter sample set, three spectrometers of varying types and resolutions were used. Information on the spectrometers used and their settings is provided in Table 1. For each sample, an 8 × 8 shot pattern was performed, providing 64 spectra per sample. A diagram of this experiment is shown in Figure 1. The measured spectra and subsequent analysis, including multivariate modeling was performed using Python along with scikit-learn and pyMCR libraries [16,17].

### 2.3. LIBS Aerosol Sample Tests

Aerosol measurements were performed using the same optical components of the previously detailed LIBS system so that models could be directly compared. Aerosols were generated by siphoning mixtures of H_2_O and D_2_O from the sample vials using a peristaltic pump into a concentric nebulizer, along with high-purity argon (AirGas, Ultra High Purity). The produced aerosols were then sent into a cyclone spray chamber, where larger droplets were removed from the stream. The larger droplets that accumulated in the spray chamber were constantly collected and removed using the same peristaltic pump in the reverse direction. The mist of fine aerosols was passed through an injector with its exit situated close to the LIBS plasma, providing a consistent aerosol stream for analysis. This aerosol sampling system is shown in the experimental graphic shown in Figure 1. This aerosol system was used to simulate aerosols that would passively form in an MSR by enabling real-time tests, changing the sample reservoir while maintaining continual LIBS measurements.

The echelle spectrometer was used for the aerosol tests with the same collection timing settings; however, only a subset of the intensified charge-coupled device (ICCD) sensor was used, to increase the spectrometer’s maximum frame rate above 10 Hz. This increase allowed data to be collected far more rapidly than the 4 Hz limit when using the entire echelle image. A total of 1000 shots were collected for each sample. The aerosol system was purged with argon between runs to prevent sample contamination. Typically, 4 mL of sample was used for each run, including 2 min of aerosol production to flush the system to minimize any sample crossover. During real-time tests, no flushing was performed between sample changes.

## 3. Results and Discussion

### 3.1. Comparison of Spectrometer Types

The first goal of this study was to demonstrate the effect that spectrometer resolution and type have on the measurement of hydrogen isotopes. When developing LIBS systems for online monitoring applications, it is important to consider the balance between cost, resolution, sensitivity, and spectral coverage when selecting a spectrometer. Here, three spectrometers have been compared: (1) a compact spectrometer that is typically used in a set of six or eight to provide full coverage of the 190–1000 nm wavelength range at a low resolution; (2) an echelle-type spectrometer equipped with an ICCD, which provides better sensitivity and moderate resolution with full wavelength coverage; and (3) a double echelle monochromator spectrometer, which provides top-tier resolution but only covers a small wavelength window. The costs associated with each spectrometer increases from option 1 to 3. Each spectrometer was used to measure the same set of calibration samples and their spectra, and their corresponding predictive capabilities were also compared.

**Figure 2:**
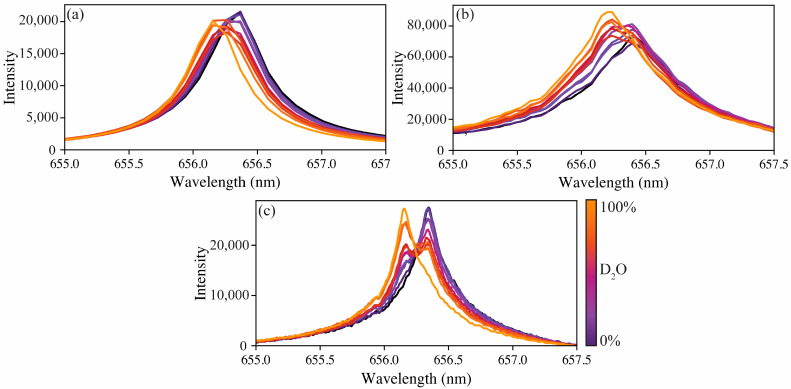
Hydrogen emission peak shift as a function of deuterium levels measured using (**a**) compact, (**b**) echelle, and (**c**) high-resolution spectrometers.

Performing LIBS directly on liquids can be difficult because of plasma shockwave propagation effects [18]. The shockwave that is generated with each laser pulse causes liquids to splash, risking the integrity of optical components. The subsequent wake leaves the liquid surface a variable distance from the optimal laser focal point. Although these issues can be counteracted through engineered sampling approaches, that is not the purpose of this study. Based on this knowledge, the initial hydrogen isotope measurements were performed by saturating glass fiber filters with aliquots of varying isotopic ratios and rastering the filter. Based on the scan pattern selected, there were 64 shots per sample. The hydrogen 656 nm emission line experiences significant Stark broadening, making its peak width extend over several nanometers. Here, Stark broadening refers to the broadening of emission lines due to the interaction between the excited species and the plasma itself. For many species, this broadening mechanism can be reduced by observing later periods in the plasma lifetime, but the hydrogen emission is short-lived, and the broadening cannot be avoided at atmospheric pressures. Single-shot emissions are typically very broad and subject to noise [9]. To overcome this issue, several shots were averaged so that the peak center could be better resolved. Figure S1 demonstrates the variation found in single-shot spectra versus the averaged spectra. Although fewer shots can be averaged, it was found that averaging all 64 shots provided the best spectra for modeling. The averaged spectra for the calibration sets for each spectrometer are shown in Figure 2.

The difference in spectral resolution is apparent when looking at Figure 2. Although all three spectrometer types can measure the peak shift, the resolution of the compact spectrometer means the protium and deuterium emissions are only separated by one wavelength step. The echelle spectrometer better captures the change from protium to deuterium; however, as anticipated, the high-resolution spectrometer captures these changes the best. The apparent peak shifts were measured to be 215, 158, and 186 pm to blue for the compact, echelle, and high-resolution spectrometers, respectively. The literature-reported peak shift from protium to deuterium is approximately 180 pm [9]. For tritium measurements, the emission peak would be shifted further towards the blue due to the increased mass difference, [19] The blue shift between tritium and deuterium is smaller than deuterium and protium (60 pm vs. 180 pm, respectively) [19]. Fortunately in an MSR, tritium and protium are would be the most abundant hydrogen isotopes so issues related to deconvoluting deuterium and tritium emissions will be limited [1].

Because the isotope shift can be seen on each of the spectrometers, the pertinent question is the ability of the spectrometers to quantify the protium-to-deuterium ratio difference. With this goal in mind, several chemometric methods were used to construct models to quantify the isotope ratio. Here, models were built using PCR, PLSR, and MCR [20,21,22,23,24].

PCR is performed by first applying principal component analysis (PCA) to reduce the dimensionality of the spectral dataset. PCA identifies orthogonal vectors that explain the variance of the independent variable (spectra) and then reduces the dataset to scores for each principal component. PCR then applies ordinary linear regression to these PCA scores [23]. PLSR considers the signal matrix (spectra) and the response matrix (isotope ratio) and transforms them into a latent space [21,22]. Here, latent variables that explain the most covariance between the signal and response matrices are solved iteratively. This process is similar to PCA; however, PCA seeks to explain the most variance in the signal matrix, whereas PLSR seeks to explain the most covariance between the signal and response matrices. In this study, only two principal components or latent variables were used for PCR and PLSR models, respectively. The third method used was MCR, which refers to the use of an alternating least squares approach to resolving the pure components from a mixture [24]. Here, MCR was applied with a non-negative constraint and a normality constraint dictating that all component concentrations sum to unity. MCR was provided with the calibration dataset and the corresponding response matrix, with which MCR determined the two spectral signals corresponding to pure protium or deuterium. MCR can be used for prediction by fitting the pure spectra components to measured spectra. Here, PCR represents the simplest modeling approach. PLSR typically provides superior prediction capabilities compared to PCR and is the most common multivariate model used for LIBS spectral analysis. Lastly, MCR represents an alternative model which provides superior interpretability by providing the pure component spectra.

To evaluate model performance, cross-validation was performed using a leave-one-out cross-validation (LOOCV) approach. Here, the model was iteratively built, leaving one sample out at a time, and then, at each iteration, the sample left out was used to test the model. The residuals for each sample while they are left out are used to calculate the root mean square error of cross-validation (RMSECV):

(1) $$RMSECV=\sqrt{\frac{\sum {\left(y_{i}-{\hat{y}}_{i}\right)}^{2}}{n}},$$

where *y_i_* is the known concentration value of the *i*th sample left out during the LOOCV iteration, ${\hat{y}}_{i}$ is the model-predicted concentration, and *n* is the number of samples [25].

**Figure 3:**
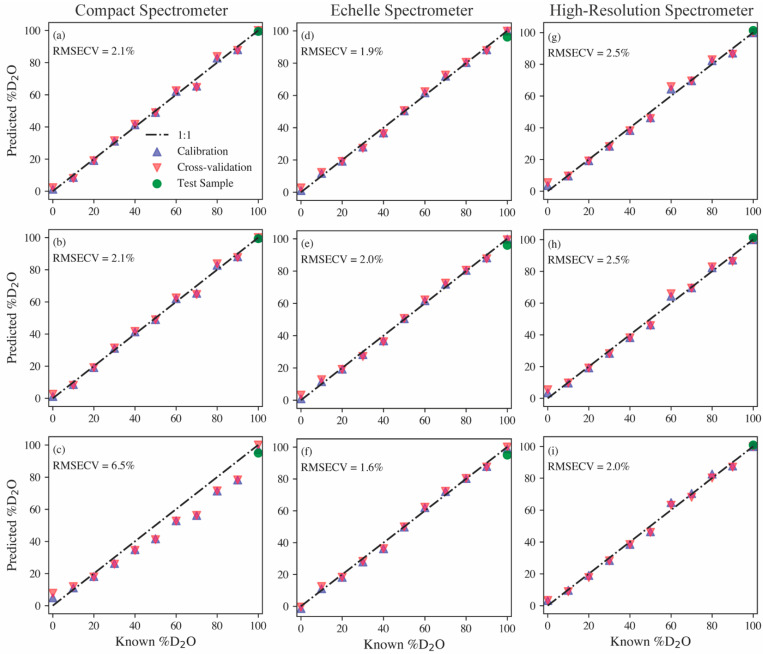
Parity plots comparing predictions with expected isotope ratios for (**a**,**d**,**g**) PCR, (**b**,**e**,**h**) PLSR, and (**c**,**f**,**i**) MCR models built using the various spectrometer types listed at the top of each column. The 1:1 line represents a perfect prediction.

Parity plots for PCR, PLSR, and MCR models built using each spectrometer type, along with their RMSECV values, are shown in Figure 3. The optimal models were built after minor preprocessing. Firstly, the high-resolution and echelle spectra were smoothed using a Savitzky–Golay filter with a first-order polynomial and a five-point window. Next, each spectrum was baseline-adjusted by subtracting the average background levels near the hydrogen peak. Lastly, each spectrum was normalized to the maximum intensity between 650 and 665 nm. The PCR and PLSR models provide relatively similar prediction performances regardless of the spectrometer type, with RMSECVs ranging from 1.9% to 2.5%. The MCR model results provide insight into how well the protium and deuterium emissions can be deconvoluted from one another based on the spectrometer. Examples of PCA loadings, PLSR regression coefficients, and the MCR pure spectral components from the echelle models are shown in the Supporting Information (Figures S2–S4). The compact spectrometer clearly struggles here with an RMSECV of 6.5%. The echelle and high-resolution spectrometer MCR models have RMSECV values on par with the previously discussed PCR and PLSR models, but the echelle MCR model performs the best out of all multivariate models with an RMSECV of 1.6%. Although Figure 2 shows the high-resolution spectrometer, providing a clearer distinguishable isotope shift, these model results indicate that the echelle spectrometer with a lower resolution can provide equivalent, if not better, quantification of the hydrogen isotope ratio.

**Figure 4:**
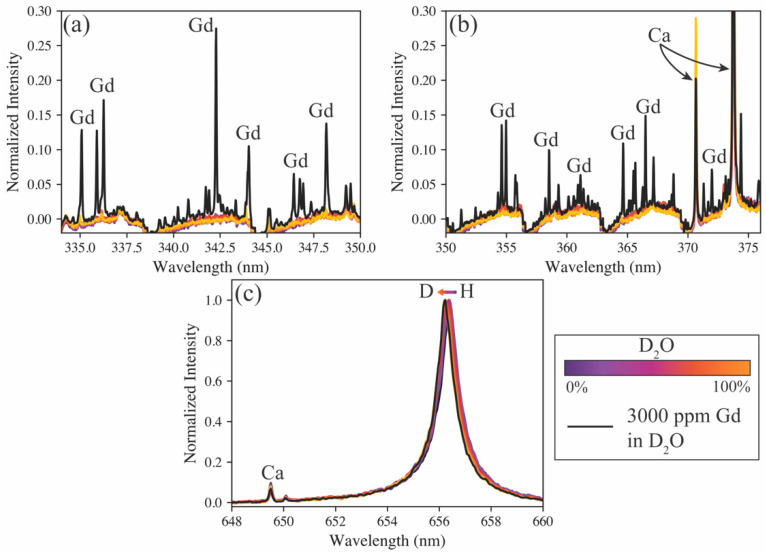
Spectrum of 3000 ppm gadolinium in D_2_O (black) compared with the protium-to-deuterium calibration spectra (purple to yellow) as measured simultaneously on the echelle spectrometer. Close up comparisons of Gd peaks against the calibration baseline are shown in (**a**,**b**). The hydrogen isotope shift is shown in (**c**).

A sample containing 3000 ppm gadolinium in D_2_O was used as a test of the models’ predictive capabilities on samples doped with other species. As seen in Figure 3 (green markers), every model estimates the hydrogen isotope ratio to be nearly 100% deuterium, as expected. This sample also provides insight into the versatility of the various spectrometers. For example, the high-resolution spectrometer may accurately monitor the hydrogen isotope ratio, but it is blind to the additional species added to the sample. Conversely, the echelle spectrometer can measure the hydrogen isotope ratio, as well as measure the added gadolinium emissions (see Figure 4). Gadolinium as the test sample addition demonstrates this capability well because of its large number of emission peaks, representative of many lanthanides, which are fission products and, in some cases, neutron poisons in MSRs [4]. The compact spectrometer has the same benefit of measuring more than just the hydrogen emissions, but in this case, the gadolinium emissions fell beyond the window of the spectrometer used. Typically, when compact spectrometers are used, several are used simultaneously; each spectrometer monitors different wavelength ranges, and their spectra can be stitched together to provide a broadband spectrum. Based on model performance and the ability to measure isotope shifts and additional species simultaneously, the echelle spectrometer was selected for further tests on aerosol samples.

### 3.2. Transferring Filter Calibration to Aerosol Measurements

The off-gas system of an MSR is a major pathway for fission product and decay daughter removal from the core. An aerosol stream is anticipated to be formed regardless of if the fuel salt is sparged or simply swept with a cover gas. Based on this expectation, it was important to demonstrate the ability to monitor hydrogen isotopes via LIBS in a continuous aerosol stream. In addition to this demonstration, the experiment poses a challenge that would be realistic for a deployed LIBS system: how a model can be effectively calibrated without running a full set of calibration samples in situ.

**Figure 5:**
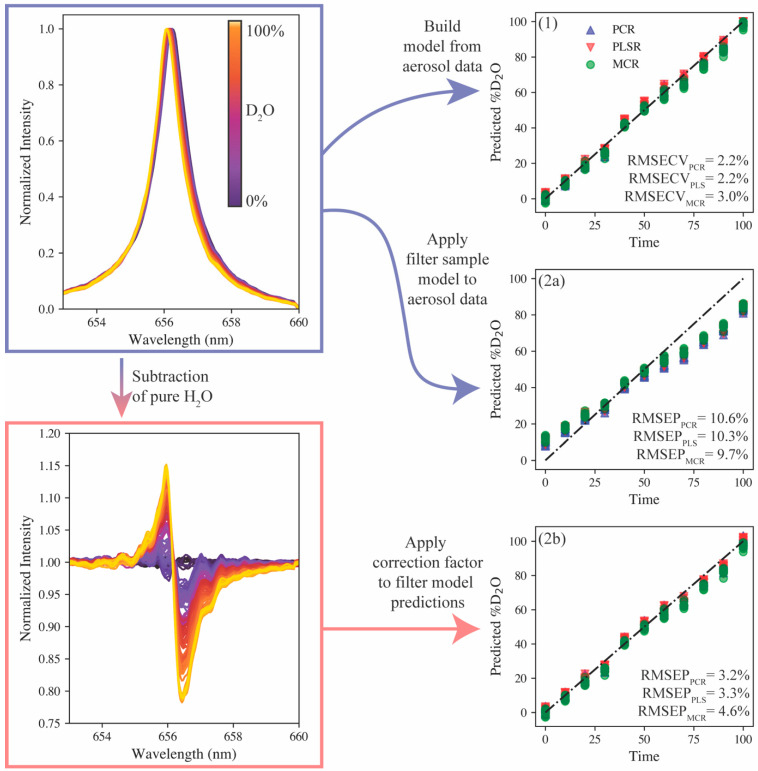
Predictive capabilities compared for (**1**) models built on aerosolized samples, (**2a**) filter sample model applied to aerosol samples, and (**2b**) modified filter sample model applied to aerosol samples with correction factor.

Firstly, the same 11 mixtures of H_2_O and D_2_O were run through the aerosol introduction system and 10 accumulate spectra (100 shots each) were collected. The normalized hydrogen emission and corresponding peak shift is shown in Figure 5. The hydrogen emission visually resembles that shown in Figure 4c; however, the broadening of the emission is larger than that seen on the filter sample set. This difference is the result of the change in matrix; the plasma formed in the aerosol stream had a greater electron density than the filter samples. The PCR, PLSR, and MCR models, with the same preprocessing steps discussed previously, were reconstructed using the aerosol spectra. The results are shown in Figure 5 (model 1). The RMSECV values were calculated to be 2.2%, 2.2%, and 3.0% for the PCR, PLSR, and MCR models, respectively. These values are larger than those using the filter samples analyzed by the echelle spectrometer, but this difference can likely be attributed to increased peak broadening that reduces the resolution of the protium and deuterium emissions.

Next, owing to the similar modeling approaches for the filter and aerosol sample sets, an attempt was made to use the chemometric models calibrated on the filter samples to predict the aerosol hydrogen isotopic compositions. The parity plot for these models’ predictions is shown in Figure 5 (model 2a). Here, because the parity plots show data not used in the calibration, the prediction error metric is the root mean square error of prediction (RMSEP). The RMSEP values range between 9.7% and 10.6%. The parity plot shows the predictions falling into tight groupings and the overall prediction series still being linear, albeit at a slope of less than 1. This is a direct consequence of the difference in sample matrix (i.e., aerosol vs. filters). While many matrix effects are reduced using preprocessing steps, the differences in Stark broadening due to differences in the plasma electron density still impact the application of the filter model onto the aerosol matrix.

To adjust the filter sample models to better predict the aerosol data, the pure protium emission spectra were subtracted from the entire filter training set, and the model was rebuilt. The aerosol data were modified in the same way before applying the model for predictions (see Figure 5). This modification forced the two datasets (calibration and prediction) to collapse onto one another, but another correction was needed to correct the slope deviation on the parity plot. For this correction, the models’ prediction of the pure deuterium sample was used to generate a correction factor to apply to all model predictions. The factor was calculated to be 1.35. The predictions of the adjusted models with the correction factor applied are shown in Figure 5 (model 2b). The RMSEP values were calculated to be 3.2%, 3.3%, and 4.6% for PCR, PLSR, and MCR, respectively. These values are greater than the model trained directly on the aerosol samples, but the PCR and PLSR models would still be valuable for quantitative monitoring. The approach of using the pure protium and deuterium aerosol samples to adjust the filter sample set model for aerosol predictions represents an 82% reduction in the volume of sample used compared with a model trained entirely on aerosol samples. This approach also represents a more feasible approach to training isotopic LIBS models for deployed systems by only needing the model end points (each pure isotope) to be tested in situ versus running a series of samples in the field.

### 3.3. Real-Time Monitoring Demonstration

As a final test and demonstration of the utility of the constructed models, a real-time test was conducted. This test leveraged the aerosol sample introduction system to pump solutions from a reservoir where the sample stream could be modified in real time by spiking, diluting, or completely changing the reservoir liquid while measuring LIBS spectra continuously. In an effort to show greater applicability to MSR effluent streams, FLiNaK salt (47:11:42 mol% LiF:NaF:KF) was dissolved in either H_2_O or D_2_O to serve as spikes during the test. The ability of LIBS to monitor each of these salt species along with the hydrogen isotope ratio truly highlights the technique’s versatility for MSR applications.

**Figure 6:**
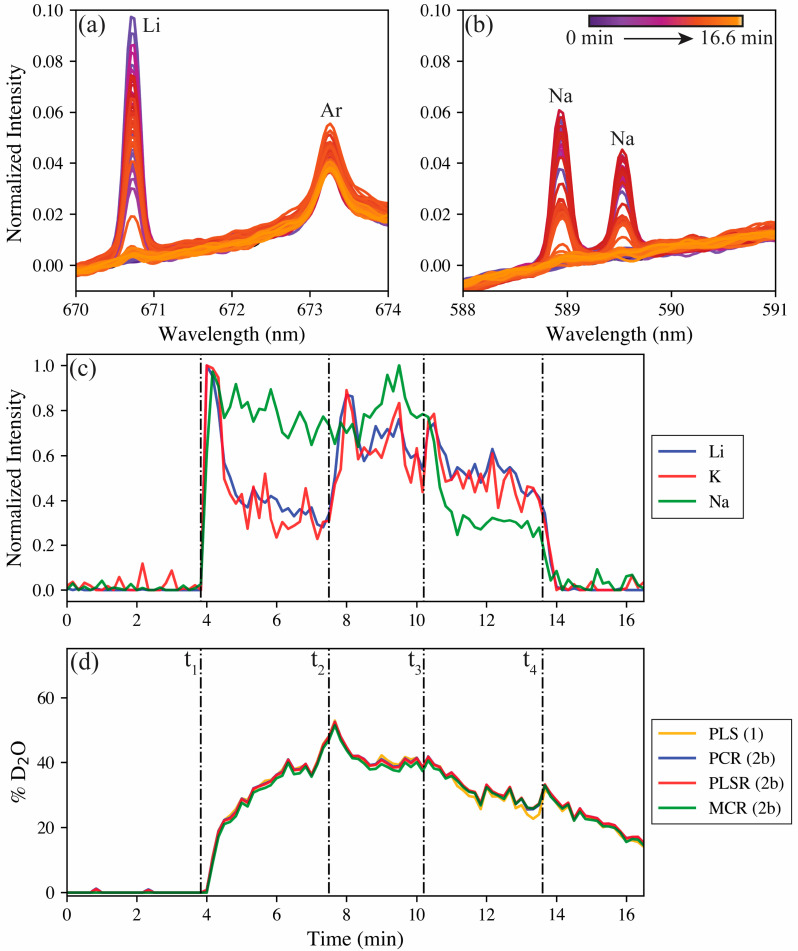
Results of the real-time test showing the spectral responses for changes in the (**a**) lithium 680.5 nm and (**b**) the sodium 589 and 589.59 nm doublet. The normalized lithium, potassium, and sodium responses to concentration spikes at *t*_1→4_ are shown in (**c**). Lastly, the factor-corrected filter model predictions (model 2b) are compared with the all-aerosol sample-trained model predictions (model 1) for the real-time measurement of hydrogen isotopes are shown in (**d**).

The real-time test was performed as follows. Firstly, pure H_2_O was run to establish a baseline, then at *t*_1_ (~3.8 min), the first spike of FLiNaK in D_2_O was added to the reservoir. This mixture was run until *t*_2_ (~7.5 min), when a spike of FLiNaK in H_2_O was added to the reservoir. At *t*_3_ (~10.2 min), a spike of pure H_2_O was added to dilute both salt species and the hydrogen isotope ratio. Lastly, at *t*_4_ (~13.6 min), the entire reservoir was replaced with pure H_2_O to return to the baseline. LIBS spectra were recorded in 100 shot accumulates at 10 Hz, providing 100 spectra over the 16.6 min. The results of the real-time test are shown in Figure 6.

The spectral response of the Li 670.8 nm emission and the Na 589 and 589.59 nm doublet are shown in Figure 6a,b. Both are nonexistent at the beginning (purple), but they rise and fall during the test from the changing reservoir before returning to the baseline at the end (bright yellow) of the test. The Ar I 763.2 nm emission remains relatively constant during the test because it originates from the aerosol carrier gas. Figure 6c shows the normalized trends for lithium (670.8 nm), sodium (589 nm), and potassium (693.9 nm, see Figure S5) over the duration of the tests, with the spike time stamps indicated by the dashed lines. Similarly, the model predictions for the hydrogen isotope ratio based on changes in peak position in the real-time test are shown in Figure 6d. No meaningful signals are seen for any of the salt species before their levels rapidly elevate following the first spike at *t*_1_. Following the initial spike, the lithium and potassium levels begin to decay. The decay of lithium and potassium can be attributed to the spike at *t*_1_ being a supersaturated mixture, where the LiF and KF crash out of solution after the initial mixture has time to settle in the reservoir. The LiF makes up 30 wt% of the FLiNaK salt composition and has a low solubility in water, so this behavior is not unexpected. The KF accounts for 59 wt% of the FLiNaK composition, so despite its high solubility in water, the amount present in the spike was above the solubility limit. The NaF accounts for far less of the FLiNaK composition (11 wt%); with this lesser amount, combined with its solubility being just under half that of KF, the NaF was expected to be nearly fully dissolved in solution, which explains its differing behavior from the other salt species. Following the D_2_O spike at *t*_1_, the hydrogen isotope ratio slowly shifts from 0% to approximately 50% deuterium, as expected, with the spike doubling the reservoir volume. The slow ingrowth of deuterium indicates that latency does exist in the aerosol sampling system with regards to the hydrogen isotopes.

The second spike contains the same concentration of FLiNaK in H_2_O. Following this spike at *t*_2_, the salt levels again spike before decaying away. Here, the hydrogen isotope ratio begins to shift back toward protium, and following the spike of pure H_2_O at *t*_3_, the hydrogen isotope ratio continues to decay at a similar rate. After this third spike, the sodium levels drop owing to being diluted by the additional H_2_O; however, the lithium and potassium levels spike and then return to a similar level as before the third spike. This spike is because of lithium and potassium salts not in solution in the reservoir now being able to dissolve to maintain the solubility limit in the reservoir. Lastly, the reservoir is completely changed to pure H_2_O at *t*_4_, the salt levels plummet to zero, and the hydrogen isotope ratio continues its decay back toward protium.

The real-time test offered the valuable opportunity to compare the multivariate models trained on the aerosol calibration samples and the filter sample models after they were corrected for aerosol measurements. The corrected PCR, PLSR, and MCR model predictions are shown in Figure 6d, overlaid with the predictions of the all-aerosol trained PLSR model. Interestingly, despite the RMSE differences between the models (see Figure 5), the disparities between model performance during the real-time test was miniscule. This difference further boasts of the opportunity offered by the ability to train LIBS models on one medium and then correct for systematic differences to transfer that model to the in-situ testing setup.

Overall, the real-time tests highlight the versatility of LIBS as an elemental/isotopic online monitoring tool for MSRs and other industrial applications. Here, LIBS monitored three independent elements that are directly tied to MSRs as typical salt species. Despite the intent to vary these salt species in similar profiles, LIBS was able to detect impacts of solubility on the true salt species composition in the aerosol stream. Meanwhile the transferred filter calibration models were able to track hydrogen isotopes with little difference compared to the all-aerosol trained system. This reaffirms that isotopic LIBS models can be built on benchtop systems and then transferred to alternative systems (e.g., engineering scale tests) by testing only two samples at the sampling destination. This will be particularly useful when these in situ calibration tests are performed under time-restrictions, such as in radioactive environments, and when the isotopes needed to train the models are limited. Being able to simultaneously track element profiles and specific isotopes (e.g., ^3^H, ^235^U, ^238^U) will be vital for MSR monitoring.

## 4. Conclusions

MSRs deviate from traditional nuclear reactors in that the fuel will be liquid and moving in and out of the core, allowing fission gases, volatile species, and aerosols to migrate into the off-gas system. This setup will necessitate advanced monitoring tools capable of handling the complex off-gas streams (e.g., radioactive species, gas/aerosol mixtures, light and heavy elements) and providing real-time information to operators/developers. This study has examined spectrometers of varying types and resolutions for measuring hydrogen isotope shifts. Additionally, this work has demonstrated the use of PCR, PLSR, and MCR to quantify this isotope shift on filter samples wetted with 10 µL droplets and aerosol streams. Finally, this work has shown a novel calibration strategy, where calibration models built using the filter sample set were modified using only two aerosol samples to be applied for aerosol monitoring. This approach offers an 82% reduction in the volume of sample needed to train the model. The models developed in this way were able to monitor hydrogen isotope ratios during real-time tests, as well as a model trained solely on aerosol samples. Future work will involve demonstrating the ability to monitor aerosol stream composition and isotopic ratios simultaneously on engineered test systems such as pumped molten salt loops, which will better reflect the conditions of an operating MSR.

## Figures and Tables

**Table 1:** LIBS spectrometer operation parameters and resolution information.

Type	Model *	Resolution (pm)	Wavelength Range (nm)	Delay (µs)	Width (µs)
Compact	Avantes, Avaspec 2048	107	501–722	3	1050
Echelle	Andor, Mechelle 5000	39	200–895	2	50
High-resolution	LTB Lasertechnik Berlin, DEMON	3.2	654–658	2	50

* Note: Avantes (Apeldoorn, The Netherlands), Andor (Belfast, Northern Ireland), LTB Lasertechnik Berlin (Berlin, Germany).

## Data Availability

Data are contained within the article and Supplementary Materials.

## Supplementary Materials

The following supporting information can be downloaded at: https://www.mdpi.com/article/10.3390/s23249797/s1.
