# Supplementary material for: Novel Calibration Approach for Monitoring Aerosol Hydrogen Isotopes Using Laser-Induced Breakdown Spectroscopy for Molten Salt Reactor Off-Gas Streams
Source: Sensors (Basel). 2023 Dec 13;23(24):9797. doi: 10.3390/s23249797 (PMC10747517; doi:10.3390/s23249797)
Supplement: Supplementary file 1 [file sensors-23-09797-s001.zip › sensors-2767457-supplementary.pdf]

sensors

MDPI

Article

Novel Calibration Approach for Monitoring Aerosol Hydrogen Isotopes Using Laser-Induced Breakdown Spectroscopy for Molten Salt Reactor Off-Gas Streams

Hunter B. Andrews[1*] and Joanna McFarlane[2]

[1] Radioisotope Science and Technology Division, Oak Ridge National Laboratory, 1 Bethel Valley Road, Oak Ridge, TN 37831, USA
[2] Nuclear Energy and Fuel Cycle Division, Oak Ridge National Laboratory, 1 Bethel Valley Road, Oak Ridge, TN 37831, USA
* Correspondence: andrewshb@ornl.gov

Supplementary Information

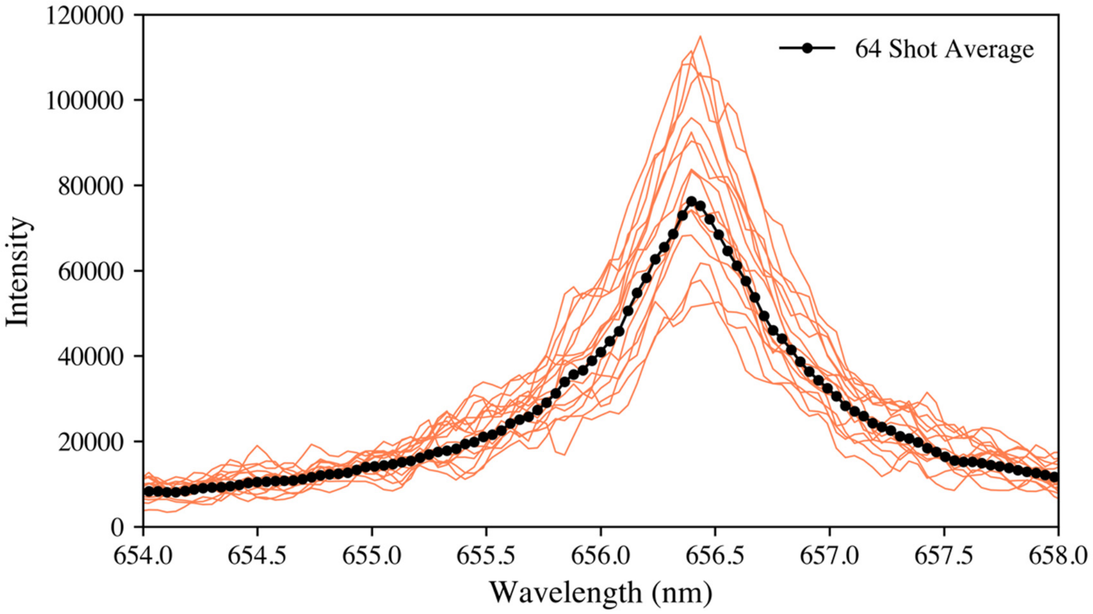

Figure S1. Single-shot spectra versus 64-shot average spectrum of 0% D_2O used to better resolve peak center.

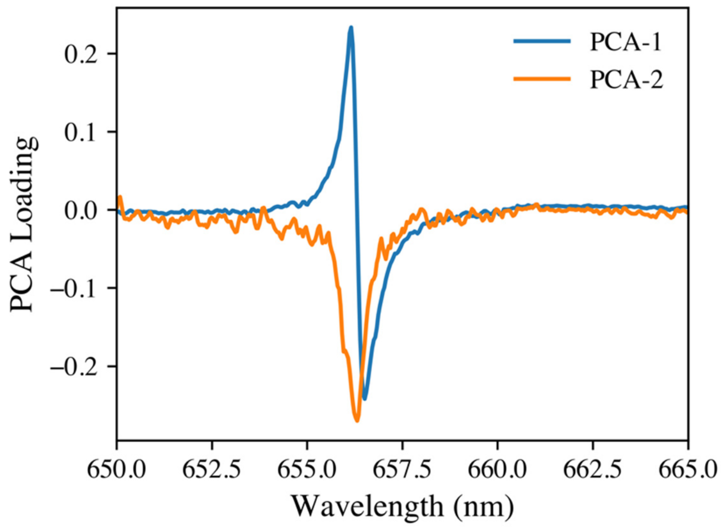

Figure S2. PCA loadings for hydrogen isotope shifts measured on the echelle spectrometer.

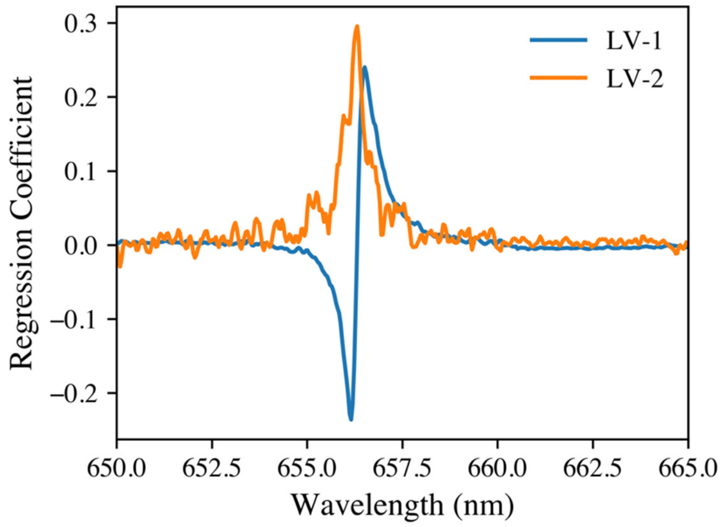

Figure S3. PLSR regression coefficients for hydrogen isotope shifts measured on the echelle spectrometer.

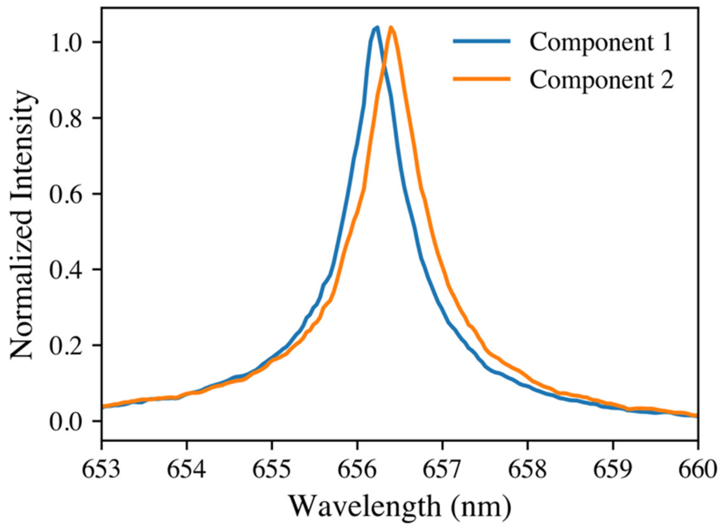

Figure S4. MCR pure spectral components for hydrogen isotope shifts measured on the echelle spectrometer.

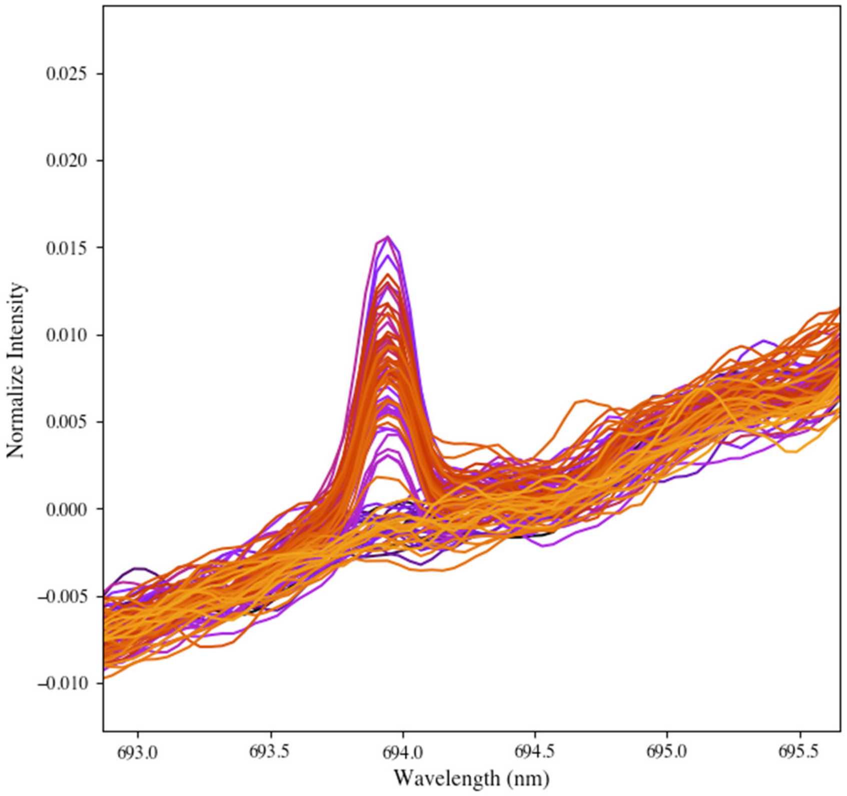

Figure S5. Potassium 693.3 nm emission over the duration of the real-time tests.
